# Genome-wide linkage analysis of QTL for growth and body composition employing the PorcineSNP60 BeadChip

**DOI:** 10.1186/1471-2156-13-41

**Published:** 2012-05-20

**Authors:** Ana I Fernández, Dafne Pérez-Montarelo, Carmen Barragán, Yuliaxis Ramayo-Caldas, Noelia Ibáñez-Escriche, Anna Castelló, Jose Luis Noguera, Luis Silió, Josep María Folch, M Carmen Rodríguez

**Affiliations:** 1 Departamento de Mejora Genética Animal, INIA, Ctra. De la Coruña km. 7, Madrid, 28040, Spain; 2 Departament de Ciència Animal i dels Aliments, Facultat de Veterinària, UAB, 08193, Bellaterra, Spain. Present address: Consorci CSIC-IRTA-UAB (Centre de Recerca en Agrigenòmica), Edifici CRAG, Campus UAB, Bellaterra, Spain; 3 Centre for Research in Agricultural Genomics (CRAG), Consortium CSIC-IRTA-UAB-UB. Edifici CRAG, Campus Universitat Autonoma Barcelona, 08193, Bellaterra, Spain; 4 Genètica i Millora Animal, IRTA Lleida, 25198, Lleida, Spain

**Keywords:** QTL, PorcineSNP60 Beadchip, Growth, Fatness, Body conformation

## Abstract

**Background:**

The traditional strategy to map QTL is to use linkage analysis employing a limited number of markers. These analyses report wide QTL confidence intervals, making very difficult to identify the gene and polymorphisms underlying the QTL effects. The arrival of genome-wide panels of SNPs makes available thousands of markers increasing the information content and therefore the likelihood of detecting and fine mapping QTL regions. The aims of the current study are to confirm previous QTL regions for growth and body composition traits in different generations of an Iberian x Landrace intercross (IBMAP) and especially identify new ones with narrow confidence intervals by employing the PorcineSNP60 BeadChip in linkage analyses.

**Results:**

Three generations (F3, Backcross 1 and Backcross 2) of the IBMAP and their related animals were genotyped with PorcineSNP60 BeadChip. A total of 8,417 SNPs equidistantly distributed across autosomes were selected after filtering by quality, position and frequency to perform the QTL scan. The joint and separate analyses of the different IBMAP generations allowed confirming QTL regions previously identified in chromosomes 4 and 6 as well as new ones mainly for backfat thickness in chromosomes 4, 5, 11, 14 and 17 and shoulder weight in chromosomes 1, 2, 9 and 13; and many other to the chromosome-wide signification level. In addition, most of the detected QTLs displayed narrow confidence intervals, making easier the selection of positional candidate genes.

**Conclusions:**

The use of higher density of markers has allowed to confirm results obtained in previous QTL scans carried out with microsatellites. Moreover several new QTL regions have been now identified in regions probably not covered by markers in previous scans, most of these QTLs displayed narrow confidence intervals. Finally, prominent putative biological and positional candidate genes underlying those QTL effects are listed based on recent porcine genome annotation.

## Background

Hundreds of QTLs have been identified in porcine species (pigQTL database), but there are still relatively few examples for which the mutations that underlie mapped QTLs have been identified [1-4]. The traditional strategy to map QTLs has been to use linkage analysis employing a limited number of microsatellite markers. These analyses usually mapped the QTLs to large intervals, 20 cM or more, which made it difficult to identify the underlying gene and mutation. The success in the positional cloning of these QTLs in domestic animals has been hampered by the absence of high-resolution linkage maps (several markers per cM) [5]. However, the arrival of genome-wide panels of SNPs makes available thousands of markers per chromosome increasing the information content and therefore the likelihood of detecting and fine map QTL regions [5,6].

The Iberian x Landrace experimental cross (IBMAP) was developed to detect QTLs for several economic traits, including growth, fatness and carcass composition [7]. The whole genome QTL scan, carried out in the F2 population using 92 microsatellite markers covering the 18 autosomes, allowed to detect three significant QTLs in SSC2, SSC4 and SSC6 [8]. Subsequent studies and even obtaining new IBMAP generations [9] have delved into knowledge of these regions. Several candidate genes, such as *LEPR, MTTP* and *FABP5,* have been analyzed reporting some successful results [10-16].

Various studies have shown the utility of high-density SNP panels for linkage analyses by providing a greater information content in comparison to microsatellites [6,17-19]. In the present study, we employed the porcine high density SNP panel, PorcineSNP60 BeadChip (Illumina), to carry out a genome QTL scan based on linkage mapping analyses using three of the generations of the IBMAP experimental population. The objective is to confirm previous QTL regions and especially identify new ones with narrow confidence intervals.

## Methods

### *Animals and Phenotypic records*

The animals and phenotypic information used in the current study belong to a F3 generation and two different backcrosses of the IBMAP experimental population [9,12]. The IBMAP F1 generation was obtained from three Iberian Guadyerbas boars and 30 Landrace sows. Six F1 boars and 73 F1sows were parents of 577 F2 pigs. Five F1 boars were mated with 25 Landrace sows obtaining 160 backcrossed animals (BC1). In addition three of the F2 boars were mated with 15 F2 sows obtaining 68 animals of the F3 generation, and finally other four F2 boars were mated with 22 Landrace sows obtaining 79 backcrossed animals (BC2). Phenotypic records used in the analyses (Table 1) included the body weight (BW) measured at 150 days of mean age (BW150), and two backfat thickness measures, one at the level of the fourth rib at 4 cm of the midline using ultrasounds at 75 kg of mean weight (BFT75) and the other taken with a rule at slaughter (BFTS). Additionally, intramuscular fat content (IMF) measured by NIRS in *longissimus dorsi* samples and weights of primary cuts (hams, HW, shoulders, SW and loin bone-in, LBW) were also registered at slaughter.

**Table 1:** Phenotypic traits recorded from the BC1 (F1 x Landrace), BC2 (F2 x Landrace) and F3 generations of the Iberian x Landrace cross

**Description**	**Trait**	**BC1generation**			**F3 + BC2 generations**		
		**N**	**Mean**	**SD**	**N**	**Mean**	**SD**
Weight at 150 days (kg)	W150d	160	79.13	10.49	161	81.69	12.64
Backfat thickness at 75 kg (mm)	BFT75	160	12.69	1.50	134	13.35	2.57
Backfat thickness at slaughter (cm)	BFTS	127	2.50	0.69	148	2.27	0.48
Intramuscular fat percentage (%)	IMF	124	2.06	0.70	147	1.08	0.56
Mean weight of hams (kg)	HW	155	10.22	1.39	148	11.44	1.68
Mean weight of shoulders (kg)	SW	155	5.43	0.80	148	4.70	0.72
Weight of bone-in loins (kg)	BLW	154	7.09	1.03	148	7.18	0.93

All animal procedures were carried out according to Spanish and European animal experimentation ethics law and approved by the institutional animal ethics committee of IRTA.

### *SNP data*

The 86 F3, 79 BC1 and 160 BC2 pigs, and their related animals from F2, F1 and F0 generations, 416 pigs in total, were genotyped with the PorcineSNP60 BeadChip (Illumina, Inc.), designed by Ramos et al. [20], using the Infinium HD Assay Ultra protocol (Illumina, Inc.). GenomeStudio software (Illumina, Inc.) was employed for visualize, edit and filter the genotyping data. Raw individual data had high-genotyping quality (call rate >0.99). The SNPs filtering was carried in our previous study [21]. Briefly, those SNPs with GenTrain Score lower than 0.85, non-Mendelian inheritance, minor allele frequency less than 0.15, located in sex chromosomes, unmapped in the Sscrofa10 assembly or showing position errors in the linkage mapping were discarded using Plink software [22]. A total of 28,633 SNPs were retained in the dataset after quality control and filtering. In addition, a selection of the most informative SNPs was carried out based in their genetic distance according to the linkage maps generated in our previous study [21]. When the genetic distance among contiguous SNPs was 0, one of them was retained as representative of the linkage group for further analyses.

### *QTL scan*

The linkage maps used for the QTL scan were obtained in Muñoz et al. [21] previous study. A joint QTL scan was performed in all BC1, BC2 and F3 animals. Moreover, two separate analyses were carried out in the BC1 and in the F3 + BC2 animals in agreement with their different parental boar origin. The QTL scans were performed with the following basic model:

(1) $$y_{\mathrm{ijk}}=S_{i}+B_{j}+u_{k}+bx_{k}+P_{\mathrm{ak}}a+e_{\mathrm{ijk}}$$

where *y*_*ijk*_ is the *ijk*^th^ observation for the analyzed trait, *S*_i_ and *B*_j_ are the systematic effects for sex (male or female) and batch (eight levels in the whole analysis, three or five levels in the analysis of BC1 or F3 + BC2 pigs), *u*_k_ is the random polygenic effect of the *k*^th^ individual, *x*_k_ is a covariable (individual age, body or carcass weight in different analyses) and *b* its respective slope, *a* is the QTL additive effect; *P*_*ak*_ is the additive coefficient calculated as *P*_*ak*_ = *Pr*(*QQ*) - *Pr*(*qq*), the probability of the *k*^th^ individual being homozygous for alleles of Iberian origin minus the probability of being homozygous of alleles of Landrace origin and *e*_ijk_ is the random residual. The infinitesimal genetic effect was treated as random, with covariance *A*σ_u_^2^*A* being the numerator relationship matrix. A single residual variance is assumed for all generations (F3, BC1 and BC2). A similar model fitting different QTL effects was used for performing complementary analyses to test the hypothesis of two QTLs mapping in different positions of the same chromosome and with effects *a*_1_ and *a*_2_ on the same trait:

(2) $$y_{\mathrm{ijk}}=S_{i}+B_{j}+u_{k}+bx_{k}+P_{a1k}a_{1}+P_{a2k}a_{2}+e_{\mathrm{ijk}}$$

Finally, joint analyses for two traits were performed to test possible pleiotropic effects of some QTL. The used model was equivalent to the basic, but here the (co)variances of the infinitesimal genetic effects are *A* ⊗ $\left(\begin{array}{cc} \sigma_{\mathrm{uy}}^{2} & \sigma_{\mathrm{uyuz}}\\ \sigma_{\mathrm{uzuy}} & \sigma_{\mathrm{uz}}^{2} \end{array}\right)$, where ⊗ denotes the Kronecker product and the subindices *y* and *z* correspond to the traits.

Likelihood ratio tests (LRT) were calculated comparing the full model and a reduced model without the corresponding QTL effect. The nominal P-values were calculated assuming a *χ*^2^ distribution of the LRT with the degrees of freedom given by the difference between the number of estimated parameters in the reduced and full models. Taking the nominal P-values resulting from the simultaneous testing, their q-values were inferred using QVALUE software [23]. The cut-off of significant QTL at the genome and chromosome level was set at q-value < 0.10. The confidence intervals (CI) were calculated at 95 % following Mangin et al. [24].

### *Gene annotation*

The physical positions of the SNPs were conducted following Sscrofa10.2 genome annotation. The SNP framing the QTL confidence intervals were used to explore gene contain in pig genome assembly 10.2. Gene annotations were retrieved from Gbrowse (http://www.animalgenome.org/cgi-bin/gbrowse/pig10/).

### *Association analyses*

Complementary association analyses were performed for specific SNPs (and haplotypes) mapped within candidate genes and included in the porcine chip. Candidate genes were identified based on their position within the QTL intervals and their functional relation with the analyzed traits. By the comparison of the SNP position with the gene position, both following Sscrofa10.2, SNPs within the candidate gene were identified and association analyses were conducted. Haplotypes were determined using Haploview software [25].

The analyses were carried out using the standard animal model:

(3) $$y_{\mathrm{ijk}}=S_{i}+B_{j}+u_{k}+bx_{k}+\lambda_{k}g+e_{\mathrm{ijk}}$$

where λ_k_ is the vector that includes an indicative variable related with the number of copies of one of the SNP or haplotype alleles, which takes 1 or -1values when the k^th^ animal was homozygous for each allele or 0 if the animal was heterozygous; *g* represented the additive effect of the SNP or haplotype.

All the statistical analyses were performed using the Qxpak v.5.1 software [26].

## Results

A total of 8,417 SNPs evenly spaced were used for the analyses. The mean distance between SNPs ranged from 0.18 cM in SSC11 to 0.33 cM in SSC6 (Table 2). The QTL scan has allowed to confirm QTL regions previously identified in the IBMAP population as well as identify new ones (Table 3) and many others at chromosome-wide significant level that are considered as suggestive (Additional file 1: Table S1).

**Table 2:** Molecular information used for the QTL scan

**SSC**	**Number of SNPs**	**Physical length Mb**	**Genetic length cM**	**Mean distance cM between SNPs**
		**(Mb)**	**(cM)**	
1	490	288.85	139.72	0.29
2	490	156.43	118.58	0.24
3	490	135.91	118.02	0.24
4	490	138.50	119.70	0.24
5	490	108.35	115.81	0.24
6	457	156.97	149.98	0.33
7	490	131.86	125.94	0.26
8	490	147.14	118.64	0.24
9	490	151.45	141.92	0.29
10	410	75.84	111.91	0.27
11	490	82.42	89.59	0.18
12	374	63.85	91.46	0.24
13	490	210.60	106.10	0.22
14	490	153.45	112.15	0.23
15	490	147.44	116.31	0.24
16	490	84.87	82.88	0.17
17	454	68.14	90.52	0.20
18	352	59.26	71.53	0.20

**Table 3:** Positions, confidence intervals and additive effects of detected significant QTL at the genome-wide level (q-value < 0.10)

**Trait**	**SSC**	**Position cM (CI)**	**a (SE)**	***P*-value**
*Whole-population*				
BFT75	4	104 (102-109)	0.901 (0.20)	8.4 x 10^-6^
	11	26 (25-27)	−0.657 (0.19)	5.7 x 10^-4^
	17	58 (56-60)	0.602 (0.19)	1.0 x 10^-3^
SW	1	104 (102-104)	−0.221 (0.07)	1.0 x 10^-3^
	4	60 (57-62)	−0.316 (0.07)	9.2 x 10^-6^
BLW	4	53 (50-54)	−0.300 (0.07)	6.0 x 10^-5^
*BC1 generation*				
BFT75	4	104 (102-109)	1.16 (0.23)	1.0 x 10^-6^
	11	26 (25-27)	−0.86 (0.22)	1.8 x 10^-4^
	14	112.5 (111-113)	0.70 (0.22)	2.0 x 10^-3^
	17	59 (56-61)	0.74 (0.23)	1.9 x 10^-3^
*F3 + BC2 generations*				
BFTS	4	82 (72-91)	0.24 (0.08)	3.0 x 10^-3^
	5	73 (71-88)	0.31 (0.09)	9.6 x 10^-4^
	6	123 (121-125)	0.32 (0.09)	3.9 x 10^-4^
	14	110 (109-113)	0.38 (0.11)	7.4 x 10^-4^
SW	2	114 (112-116)	0.45 (0.11)	2.8 x 10^-4^
	4	61 (60-64)	−0.35 (0.08)	1.0 x 10^-5^
	6	135 (134-136)	−0.34 (0.11)	2.0 x 10^-3^
	9	111 (106-113)	−0.23 (0.08)	2.7 x 10^-3^
	13	49 (47-53)	−0.28 (0.09)	3.0 x 10^-3^
BLW	2	116 (114-117)	0.74 (0.16)	1.3 x 10^-5^

The joint scan of both populations (BC1, F3 + BC2) revealed QTL regions in ten of the 18 autosomes (Table 3 and Additional file 1: Table S1). Six of which were significant to the genome-wide level: three QTLs for BFT75 in SSC4, SSC11 and SSC17, two for SW in SSC1 and SSC4 and one for BLW in SSC4 (Table 3). A complementary analysis was carried out in order to test possible pleiotropic effects of the SSC4 QTL for SW and BLW (Table 3). The results showed a significant pleitropic QTL (*P*-value = 5.7 x 10^-6^) at 60 cM with additive effects on these traits (-0.24 ± 0.05 kg and -0.28 ± 0.07 kg, respectively).

The QTL detection analyses carried out in the BC1 generation revealed QTL regions in 14 of the 18 porcine autosomes (Tables 3 and Additional file 1: Table S1), four of which were significant to the genome-threshold level. These genome–wide QTLs were identified in SSC4, SSC11, SSC14 and SSC17 for BFT75 trait (Table 3).

The QTL scan in the F3 + BC2 generations showed QTL regions in 11 of the 18 porcine autosomes (Tables 3 and Additional file 1: Table S1). Ten of these were significant to the genome-wide level: for BFTS in SSC4, SSC5, SSC6 and SSC14, for SW in SSC2, SSC4, SSC6, SSC9 and SSC13 and for BLW in SSC2 (Table 3). A complementary analysis was carried out in order to test pleiotropic effects of the SSC2 QTL for SW and BLW (Table 3). The results showed a significant pleitropic QTL (*P*-value = 1.1 x 10^-4^) at 116 cM with additive effects on these traits (0.40 ± 0.11 kg and 0.74 ± 0.16 kg, respectively).

**Figure 1:**
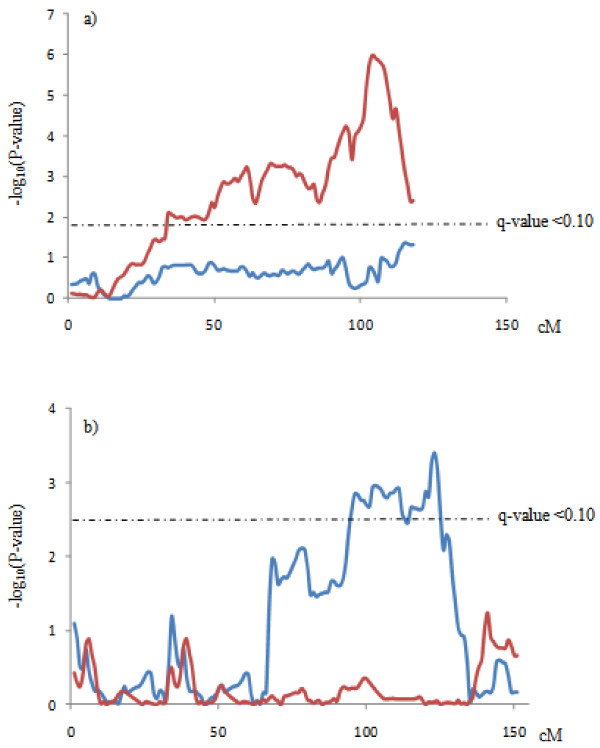
**QTL significant profiles in the separate population analyses of SSC4 for BFT75 (a) and SSC6 for BFTS (b).** Red lines represent the QTL significant profiles in the BC1 generation and blue lines in the F3 + BC2 generations.

**Figure 2:**
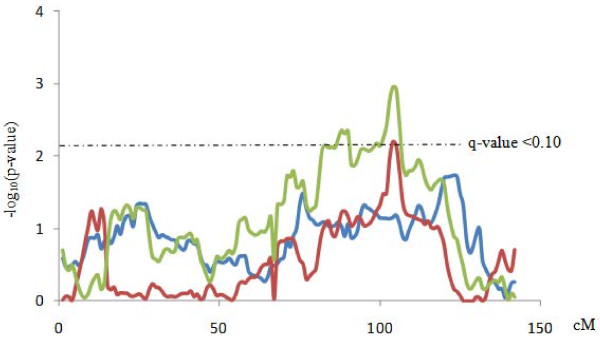
**QTL significant profiles in SSC1 for SW in the separate F3 + BC2 and BC1 and in the whole population.** Green line represents the QTL significant profile in the whole population, red line in the BC1 generation and blue line in the F3 + BC2 generations.

The separate analyses, using the same set of SNPs markers, evidenced differences between the populations. Examples of these differences are shown in Figure 1. The joint analyses allowed to capture some of the QTLs identified in the separates analysis (in SSC4, SSC11 and SSC17 for BFT75). Nevertheless, a QTL in SSC1 for SW reached the genome-wide significance in the joint analysis but not in the separate ones (Figure 2).

## Discussion

The genome wide association study (GWAS) is the approach widely used for the analysis of high density SNP data. In the present study a classical QTL scan, based on the parent line origin assuming alternative alleles fixed in each of the parental populations, has been considered appropriate for the QTL detection analysis in agreement with the experimental design. The QTL scan using this high density panel of 8,417 SNPs has allowed the confirmation of QTL regions previously identified in the IBMAP population. Moreover, new QTLs have been detected, despite using a limited number of animal data, in regions probably not covered by the limited number of microsatellite markers used in previous studies.

Two different QTL analyses were carried out, a joint QTL scan and two separate analyses in agreement with the different parental boar origin of the generations. The separate analyses evidenced the differences between populations regarding the expected QTL genotypes and the random sampling of the QTL alleles in F1 and F2 boars. While only Qq and qq genotypes, coming from the F1 boars, are expected for the QTLs in the BC1 animals, the three possible QTL genotypes (QQ, Qq and qq, coming from the F2 boars) are possible in BC2 and F3 pigs. In addition, the F2 boars used for F3 and BC2 were selected conditioned on their potential genotypes for different QTL (mainly the QTL for growth and fatness in SSC4 and SSC6); however no selection could be done for the F1 boars used for BC1. These differences between populations are reflected in the results obtained. The joint analyses allowed to capture some of the QTLs identified in the separates analysis (in SSC4, SSC11 and SSC17 for BFT75) but not most of them. Nevertheless, other QTL, the one detected in SSC1 for SW, reached the genome-wide significance in the joint analysis but not in the separate one, indicating a gain of detection power with the increase of the record number for this QTL.

**Figure 3:**
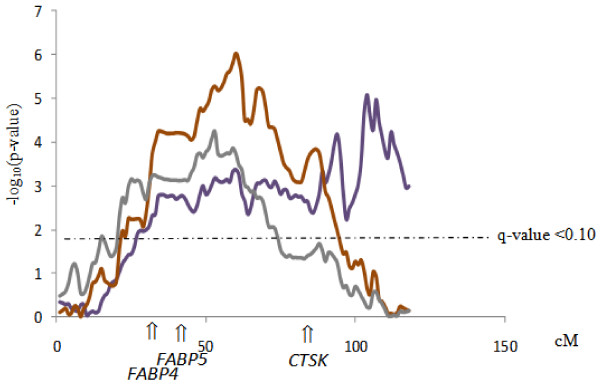
**QTL significant profiles in SSC4 for BFT75, SW and BLW traits in the whole-population analysis.** Purple line represents the QTL significant profile for BFT75, brown line for SW and gray line for BLW.

The most significant QTL region identified in the present study corresponded to the detected in SSC4 for BFT75, SW and BLW in the joint analysis. The likelihood profiles, shown as –log_10_(*P*-value), showed the presence of at least two QTL regions in SSC4, with a maximum located around 53-60 cM position for SW and BLW and around 104 cM for BFT75 (Figure 3). The first QTL region as well as the effects agree with one of the most relevant QTL for growth and fatness described in the IBMAP material for growth and fatness [8,9], overlapping the known *FAT1* region [27,28]. Moreover, a complementary analysis revealed that this first QTL region presented pleotropic effects on SW, BLW and BFTS. However, the second QTL region, around 104 cM affecting BFT75 has been identified for the first time in the IBMAP material, but it has been already described in other populations (Table 4). In addition, the QTL significant profile of SSC4 scan for BFT75 in the BC1 may indicate another potential QTL region around 75 cM (Figure 3). Nevertheless, a complementary analysis employing a model with two QTL *vs* one single QTL did not allow to detect this possible secondary QTL. Another of the most relevant QTL regions for growth and fatness previously identified in the IBMAP experimental population was located around *LEPR* gene in SSC6 [10]. In the present analyses, this QTL has also been detected but only in the F3 + BC2 population (Figure 1). The QTL effects agree with the previously described for backfat thickness and shoulder weight. The *Q* Iberian allele led to an increase of the backfat and a decrease of the shoulder weight. This QTL could not be detected in the BC1, probably due to the lack of QTN segregation in this animal material. In fact, a previous study of the putative causal mutation of this QTL, *LEPR* c.1987 C > T, on this BC1 material, could not reveal significant associations due to the scarcity of some genotypes [13]. The remaining significant genome-wide QTL regions identified in the porcine chromosomes 1, 2, 5, 9, 11, 13, 14 and 17 in one or both populations have been previously associated with growth, fatness and conformation traits in the porcine species (Table 4). However, they were not detected in previous IBMAP scans, except the QTL in SSC9 for SW and in SSC14 for BFT but at suggestive signification level [8]. No genome-wide QTLs could be detected for W150d, IMF and HW traits, probably due to the limited size of the analyzed data, as several ones were detected to chromosome-wide significance level (Additional file 1: Table S1).

**Table 4:** **New QTL regions identified in the present study*vs*QTLdb and GWAS analysis performed by Fan et al. study**[29]

**SSC**	**Position cM**	**Trait QTLdb**	**Reference**
1	102-104	BFT	[29-31]
		BW	[32]
		ABDF	[33]
		ADG	[32,34]
2	112-117	ADG	[32,35]
4	102-109	10RIBBFT	[36]
5	71-88	BFT	[29,32,37-39]
		BYLEAN	[40]
		ADG	[41]
9	106-113	BW	[42]
		BFT	[37,42]
11	25-27	BFT	[38,43]
		BELLYWT	[30]
13	47-53	BFT	[44,45]
		ADG	[43]
14	109-113	BELLYWT	[37]
		BFT	[29]
17	56-61	ADG	[46]
		BW	[46]
		FATCP	[47]
		FEEDCON	[48]
		HW	[48]

BFT: backfat thickness, BW: body weight, ABDF: abdominal fat weight; ADG: average daily gain; 10RIBBFT: Backfat at tenth rib; BYLEAN: Belly meat content; BELLYWT: Belly weight; FATCP: fat-cuts percentage; FEEDCON: Feed conversion ratio; HW: ham weight.

Most of the significant QTL regions identified in the present study displayed CI shorter than 5 cM (Table 3), which should facilitate the identification of suitable candidate genes. Thanks to the huge effort of Swine Genome Sequencing Consortium on porcine genome assembly and SNP annotation, a refined search of positional candidate genes could be carried out using Gbrowse tool (Table 5). It should be noted that porcine gene annotation is still scarce, several genes are projected but not annotated; nevertheless gene position within the QTL is more likely to be correct that those inferred from comparative humane-porcine mapping used in previous works. In addition, many annotations discrepancies exist between databases, most likely due to mapping differences between porcine genome versions. In the current study we have mainly used, assuming as more reliable, the latest Sscrofa10.2 genome version for gene annotation. The following paragraphs expose a discussion of the positional and biological putative candidate genes for each of the genome-wide significant QTL regions, according to current porcine gene annotation and available biological information.

**Table 5:** Annotated genes within the confidence intervals of the new QTLs identified in the present study according to Gbrowse tool

**SSC**	**Position**		
	**Linkage (cM)**	**Sscrofa10.2 (Mb)**	**Official gene symbol**
1	102-104	232.67-240.39	*TYRP1,****PTPRD***
2	112-117	150.90-158.32	*ARHGAP26, NR3C1, LARS, RBM27, POU4F3, SLC6A7, CSF1R, HMGXB3,****PPARGC1B****, PPP2R2B, DPYSL3, JAKMIP2, FBXO38, HTR4, SH3TC2, AFAP1L1, ARHGEF37, CSNK1A1, PDGFR*
4	102-109	129.65-134.38	***VCAM1****, SLC35A3,****AGL****, PALMD, DPYD, CNN3, FRRS1, SNX7, PTBP2, ACN9, RNDD3, TMEM56, A4H2R6*
5	71-88	69.45-83.72	*TEAD4, FOXM1, FKBP4, WASH1,SLC6A3, KDM5A, WNK1, RAD52, ERC1,****ADIPOR2****, CACNA2D4, ATP6U1E1, BCL2L13, BID, MICAL3, USP18, CPNE8, KIF21A, ABCD2, IRAK4, TMEM117, ANO6, ARID2, SCAF11, SLC38A1, SLC34A4,****VDR****, TMEM106C, COL2A1, SENP1, LALBA, APLP2, ALDH1L2, TXNRD1*
9	106-113	120.96-127.05	*NOBOX, TMEM183A,****MYOG****, MYBPH, CHI3L1, CHIT1, PRRC2C, MYOC, VAMP4, METTL13, PIGC,****FASLG****, TNFRSF4*
11	25-27	26.44-27.89	*KBTBD6, MTRF1, PCDH8*
13	47-53	83.36-91.03	*AMOTL2, ANAPC13, MSL2, PCCB, STAG1, TMEM22,****NCK1****, RASA2, GRK7, XRN11, CLDN18, ESYT3, CEP70, FAIM, PIK3CB, FOXL2, COPB2, TRPC1, RBP2, RBP1, CLSTN2, TRIM42, SLC25A36, ACPL2, ZBTB38*
14	109-113	149.48-153.59	*MGMT, GLRX3, PWWP2B, INPP5A, KNDC1, ADAM8, ZNF511,****CYP2E1***
17	56-61	39.58-42.08	*TRIB3, TBC1D20, BD129, BD125, REM1,****ID1****, BCL2L1, MYLK2, TPX2, TM9SF4, PLAGL2, POFUT1, ASXL1, DNMT3B, MAPRE1, SUN5, BP1F cluster, CDK5RAP1, SNTA1, CBFA2T2*

In bold appears the most prominent putative biological candidates.

The CI (102-104 cM, 232.7-240.4 Mb) of the SSC1 QTL for SW includes 11 protein-coding genes, however only two are annotated to known genes (Table 5). Although no study in porcine has been focused on *PTPRD* gene, studies in human suggest that *PTPRD* gene could play a relevant role in glucose homeostasis and insulin sensitivity [49].

Within the pleiotropic CI (112-117 cM, 150.9-158.3 Mb) of SSC2 QTL for SW and BLW, there are 63 protein-coding genes, 19 out of them are annotated to known genes (Table 5). Among these, *PPARGC1B* constitutes a strong candidate, although it has never been studied in porcine species. PPARGC1B belongs to the PGC-1 family, which act as coactivators in the dysregulation in diseases such as diabetes, obesity and cardiomyopathy in humans [50].

As it was mentioned before, the SSC4 QTL for fatness and conformation traits was identified in previous IBMAP scans [7,8]. In addition, subsequent studies have aimed to deepen the knowledge of this region and some candidate genes (Figure 3) have been analyzed reporting different results [15,16,51-53]. In the present study, apart from that region around 60 cM, another QTL region for BFT75 has been identified around 104 cM position. Within the CI of this second QTL (102-109 cM, 129.7-134.4 Mb) there are 18 protein-coding genes, 12 out of them are annotated to known genes (Table 5), highlighting the *AGL* and *VCAM1* genes as powerful biological candidates underlying the QTL effects. Han et al. [54] study revealed associations of an indel polymorphism in the *AGL* gene with growth, fatness and carcass traits in an F2 population crossbred Landrace and Jeju (Korea) Black pigs. Recently, Fontanessi et al. [55] study revealed associations of one SNP in *VCAM1* gene with backfat thickness in Italian Large White pigs.

The SSC5 QTL for BFTS showed the largest CI (71-88 cM, 69.5-83.7 Mb), including more than 100 protein-coding genes, 34 of which are annotated to known genes (Table 5). Among the long list of putative candidates, *ADIPOR2* and *VDR* genes highlight as powerful biological candidates, although they have never been studied as candidate gene for fatness in porcine species. The ADIPOR2 mediates the increased AMPK and PPAR-alpha ligand activities, as well as fatty acid oxidation and glucose uptake by adiponectin [56]. Human studies suggest that VDR may function as a determinant of muscle strength, fat mass and body weight [57].

The *LEPR* gene is the most powerful candidate underlying the QTL for fatness and conformation traits mapped in SSC6 in the F3 + BC2 generation. In fact, a highly significant association of a polymorphism located in exon 14, *LEPR* c. 1987 C > T, with growth and fatness has been previously found in several generations of the IBMAP population [11,12]. These effects have been also confirmed in other porcine populations [58-61]. Moreover, functional studies have revealed differences in the LEPR mRNA expression levels in hypothalamus conditional on *LEPR* c.1987 C > T genotype [13] in agreement with the potential causal effect of this QTL on growth and fatness.

The CI (106-113 cM, 121.0-127.1 Mb) of the SSC9 QTL for SW includes 44 protein-coding genes, 13 out of them are annotated to known genes (Table 5). Among the potential list of candidates, the *MYOG* gene plays an essential role in the development and differentiation of muscle. Moreover, studies in porcine species have investigated the associations of *MYOG* polymorphisms with carcass composition and meat quality in pigs evidencing significant associations [62,63]. Also, *FASL* gene has been implicated in skeletal myogenesis [64].

The SSC11 QTL for BFT75 showed one of the shortest CI (25-27 cM, 26.4-27.9 Mb). Within this region only nine protein-coding genes are projected, three of which are annotated to known genes (Table 5), however there is not a feasible candidate as the biological function of these genes have not been elucidated yet.

The CI (109-113 cM, 149.5-153.6 Mb) of SSC14 QTL for live backfat deposition and at slaughter includes 43 protein-coding genes. The *CYP2E1* gene appears among the eight annotated to known genes. The *CYP2E1* has been widely studied in pigs regarding boar taint [65-67], however, its relation to porcine lipid metabolism and fatness has never been explored, even if its key role in obesity and insulin resistance phenotypes has been showed in rodents and humans [68,69].

The SSC13 QTL for SW showed a CI of 7 cM (47-53 cM, 83.4-91.0 Mb). Within this interval 64 projected protein-coding genes are mapped, 25 of which are already annotated to know genes (Table 5). Among them, *NCK1* gene is found as a functional candidate. This gene encodes for a protein implicated in regulating the unfolded protein response, which secondary to obesity impairs glucose homeostasis and insulin actions [70].

Finally, the CI (56-61 cM, 39.6-42.1 Mb) of SSC17 QTL for BFT75 contains 64 coding-protein projected genes, 28 of which are annotated to known genes (Table 5). Among the annotated gene list, the *ID1* gene highlights as biological candidate to underlay the QTL effects. Studies in mice suggest that ID1 is a negative regulator of insulin secretion, playing an essential role in the etiology of glucose intolerance, insulin secretory dysfunction, and β-cell dedifferentiation under conditions of increased lipid supply [71].

Additionally, we noted that some SNPs within five genes (*PTPRD, AGL, VCAM1, VDR*, *FASL* and *CYP2E1*) considered positional and biological candidates, as it is mentioned in the previous paragraphs, are contained in the PorcineSNP60 BeadChip, according to Sscrofa10.2 annotation. Therefore, these SNPs were tested to underlay the corresponding QTL effects in association analyses (Table 6). The main results of the SNP association were for two of the *AGL* SNPs with BFT75 in the BC1 generation. Even more, a haplotype analysis of the two *AGL* SNPs (haplotypes: ASGA0022526G-ALGA0028692C (H1), ASGA0022526A-ALGA0028692G (H2) and ASGA0022526A-ALGA0028692C (H3)) revealed higher significant effects than the single SNP analyses (*P*-value = 4.6 x 10^-6^). The H1 haplotype showed the strongest effect (1.02 ± 0.22 mm). These SNPs are located in non-coding regions and they are likely in linkage disequilibrium with the causative mutation underlying the QTL effects in SSC4.

**Table 6:** Results of the association analyses of the SNPs that are contained in the PorcineSNP60 BeadChip and mapped within candidate genes underlying the QTL effects in SSC1, 4, 5, 9 and 14

**Gene**	**SNP**	**Minor alelle frequency**	**g (SE)**	***P*-value**
*QTL in SSC1 for SW (Whole population)*				
*PTPRD*	ASGA0005690	0.27	−0.01 (0.06)	0.988
	INRA0005932	0	--	--
*QTL in SSC4 for BFT75 (BC1 generation)*				
*AGL*	ASGA0022526	0.19	1.15 (0.51)	0.025
	ASGA0022527	0.04	--	--
	ALGA0028692	0.27	0.87 (0.22)	1.8 x 10^-4^
*VCAM1*	DIAS0002972	0.18	0.84 (0.39)	0.033
*QTL in SSC5 for BFTS (F3 + BC2 generations)*				
*VDR*	DIAS0001339	0.43	−0.01 (0.07)	0.881
	MARC0076697	0	--	--
*QTL in SSC9 for SW (F3 + BC2 generations)*				
*FASL*	DBUN0000737	0.33	−0.03 (0.07)	0.622
	H3GA0028097	0	--	--
*QTL in SSC14 for BFT75 (BC1)*				
*CYP2E1*	UMB10000045	0.14	−0.12 (0.39)	0.755

## Conclusions

The arrival of the high-density SNP panels makes available high-resolution linkage maps increasing the information content for the successful QTL identification. In the current study, the use of the PorcineSNP60 BeadChip has allowed to detect significant QTL for fatness and yield cuts in ten autosomes (SSC1, SSC2, SSC4, SSC5, SSC6, SSC9, SSC11, SSC13, SSC14 and SSC17). Two of the QTL regions, in SSC4 and SSC6, had been previously identified in the same animal material, however, the remaining ones were not previously detected probably due to the limited number of microsatellite markers employed in those scans. Moreover, most of the significant QTL regions displayed narrow CI making easier the selection of candidate genes. Finally, prominent putative biological and positional candidate genes underlying those QTL effects are listed based on recent porcine genome annotation.

## Abbreviations

QTL, Quantitative trait loci; SNP, Single nucleotide polymorphism; LEPR, Leptin receptor; MTTP, Microsomal triglyceride transfer protein; FABP5, Fatty acid binding protein 5; TYRP1, Tyrosinase-related protein 1; PTPRD, Protein tyrosine phosphatase, receptor type, D; PPARGC1B, Peroxisome proliferator-activated receptor gamma, coactivator 1 beta; AGL, Amylo-alpha-1, 6-glucosidase, 4-alpha-glucanotransferase; VCAM1, Vascular cell adhesion molecule 1; ADIPOR2, Adiponectin receptor 2; VDR, Vitamin D (1,25- dihydroxyvitamin D3) receptor; AMPK, Protein kinase, AMP-activated, beta 1 non-catalytic subunit; MYOG, Myogenin (myogenic factor 4); FASL, Fas ligand (TNF superfamily, member 6); CYP2E1, Cytochrome P450, family 2, subfamily E, polypeptide 1; NCK1, NCK adaptor protein 1; BPIF, BPI fold containing family C; ID1, Inhibitor of DNA binding 1, dominant negative helix-loop-helix protein.

## Competing interests

The authors declare that they have no competing interests.

## Authors’ contributions

AIF, JMF, JLN, MCR and LS conceived and designed the experiment. MCR and AIF performed the data analysis and drafted the manuscript. JLN, NIE, AIF, MCR, LS and JMF collected the samples and records. AC performed the SNP genotyping, CB, AIF and YR performed the SNP filtering. DPM and AIF performed the SNP probe and gene annotation. All authors read and approved the final manuscript.

## Supplementary Material

Additional file 1 **Table S1.** Positions and additive effects of significant QTL at the chromosome-wide level (q-value < 0.10). Click here for file
