# Supplementary material for: Genome-wide linkage analysis of QTL for growth and body composition employing the PorcineSNP60 BeadChip
Source: BMC Genet. 2012 May 20;13:41. doi: 10.1186/1471-2156-13-41 (PMC3432624; doi:10.1186/1471-2156-13-41)
Supplement: Additional file 1 — Table S1. Positions and additive effects of significant QTL at the chromosome-wide level (q-value < 0.10). [file 1471-2156-13-41-S1.docx]

Table 1 – Positions and additive effects of significant QTL at the chromosome-wide level (q-value < 0.10)

| Trait | SSC | Position cM | a (SE) | *P*-value |
| --- | --- | --- | --- | --- |
|  |  |  |  |  |
| *Whole-population* | | | |  |
| W150d | 4 | 58 | -3.38 (1.05) | 1.0 x 10^-3^ |
|  | 7 | 5 | 3.23 (1.08) | 2.8 x 10^-3^ |
|  | 10 | 27 | -4.16 (1.22) | 7.0 x 10^-3^ |
| BFT75 | 5 | 103 | -0.47 (0.19) | 1.3 x 10^-2^ |
| BFTS | 4 | 74 | 0.20 (0.07) | 4.1 x 10^-3^ |
|  | 12 | 91 | -0.27 (0.07) | 1.9 x 10^-4^ |
|  | 14 | 85 | 0.25 (0.07) | 8.2 x 10^-4^ |
|  | 16 | 21 | -0.23 (0.08) | 6.2 x 10^-3^ |
| HW | 1 | 105 | -0.32 (0.10) | 1.6 x 10^-3^ |
|  | 4 | 59 | -0.29 (0.10) | 4.4 x 10^-3^ |
|  |  |  |  |  |
| *BC1 generation* | |  |  |  |
| W150d | 1 | 1 | -4.51 (1.24) | 3.3 x 10^-4^ |
|  | 2 | 84 | 3.18 (1.31) | 1.5 x 10^-2^ |
|  | 4 | 58 | -3.27 (1.30) | 1.3 x 10^-2^ |
|  | 7 | 120 | 3.67 (1.25) | 3.7 x 10^-3^ |
|  | 9 | 133 | 4.34 (1.26) | 6.8 x 10^-4^ |
|  | 10 | 54 | -4.49 (1.29) | 5.9 x 10^-4^ |
|  | 13 | 88 | 3.68 (1.21) | 2.5 x 10^-3^ |
| BFTS | 12 | 91 | -0.37 (0.11) | 8.3 x 10^-4^ |
|  | 14 | 85 | 0.43 (0.11) | 1.5 x 10^-4^ |
|  | 15 | 64 | 0.30 (0.12) | 9.6 x 10^-3^ |
|  | 16 | 46 | -0.28 (0.11) | 4.7 x 10^-3^ |
| HW | 1 | 96 | -0.26 (0.09) | 5.3 x 10^-3^ |
|  | 2 | 33 | -0.22 (0.09) | 2.3 x 10^-2^ |
|  | 13 | 47 | 0.23 (0.09) | 1.4 x 10^-2^ |
| SW | 4 | 60 | -0.17 (0.05) | 2.1 x 10^-3^ |
|  | 6 | 3 | -0.17 (0.05) | 1.5 x 10^-3^ |
|  | 15 | 14 | -0.14 (0.05) | 7.9 x 10^-3^ |
|  | 17 | 24 | -0.15 (0.05) | 4.6 x 10^-3^ |
| BLW | 4 | 53 | -0.27 (0.09) | 2.1 x 10^-3^ |
|  | 15 | 91 | -0.22 (0.09) | 1.4 x 10^-2^ |
|  | 17 | 13 | -0.22 (0.08) | 9.7 x 10^-3^ |
|  |  |  |  |  |
| *F3+BC2 generations* | | | |  |
| W150d | 6 | 111 | 5.3 (1.85) | 4.4 x 10^-3^ |
|  | 9 | 86 | -5.02 (1.75) | 4.6 x 10^-3^ |
|  | 12 | 3 | 6.92 (1.99) | 7.8 x 10^-4^ |
| ETD75 | 8 | 45 | 1.05 (0.33) | 2.9 x 10^-3^ |
|  | 13 | 31 | 1.25 (0.34) | 5.0 x 10^-4^ |
|  | 18 | 57 | -1.43 (0.44) | 2.0 x 10^-3^ |
| BFTS | 11 | 68 | 0.21 (0.08) | 1.1 x 10^-2^ |
|  | 12 | 50 | 0.28 (010) | 5.0 x 10^-3^ |
| IMF | 2 | 115 | -0.45 (0.12) | 3.2 x 10^-4^ |
| HW | 2 | 115 | 0.99 (0.26) | 5.4 x 10^-4^ |
|  | 4 | 61 | -0.46 (0.18) | 1.1 x 10^-2^ |
|  | 5 | 6 | -0.68 (0.26) | 7.8 x 10^-3^ |
|  | 13 | 18 | -0.57 (0.20) | 4.0 x 10^-3^ |
| BLW | 4 | 63 | -0.39 (0.12) | 1.6 x 10^-3^ |
|  | 9 | 111 | -0.39 (0.11) | 6.2 x 10^-4^ |
